# Imaging manifestations of von Hippel-Lindau disease: an illustrated guide focusing on abdominal manifestations

**DOI:** 10.1590/0100-3984.2021.0121-en

**Published:** 2022

**Authors:** Daniel Alvarenga Fernandes, João Luiz Veloso Mourão, Juliana Ávila Duarte, Mariana Dalaqua, Fabiano Reis, Nelson Marcio Gomes Caserta

**Affiliations:** 1 Department of Radiology, Faculdade de Ciências Médicas da Universidade Estadual de Campinas (FCM-Unicamp), Campinas, SP, Brazil; 2 Department of Radiology and Diagnostic Imaging, Hospital de Clínicas de Porto Alegre (HCPA), Porto Alegre, RS, Brazil; 3 Hôpitaux Universitaires de Genève, Service de Radiologie, Geneva, Switzerland

**Keywords:** von Hippel-Lindau disease/diagnostic imaging, Carcinoma, renal cell, Pancreatic neoplasms, Pheochromocytoma, Doença de von Hippel-Lindau/diagnóstico por imagem, Carcinoma de células renais, Neoplasias pancreáticas, Feocromocitoma

## Abstract

Von Hippel-Lindau (VHL) disease is a monogenic autosomal dominant disorder with germline mutations of the VHL anti-oncogene on the short arm of chromosome 3 (3p25-26). It affects 1:36,000-50,000 individuals, with a penetrance greater than 90% at 65 years of age. Although of variable onset and presentation, with pleiotropism even among members of the same family who share a specific mutation, VHL disease usually manifests initially in young adults. It predisposes to the development of benign and malignant tumors of the central nervous system (CNS) and visceral organs. The clinical diagnosis of VHL disease can be made in the following circumstances: a) in patients with a family history of the disease and at least one of the tumors characteristic of it (e.g., retinal or CNS hemangioblastomas, clear cell renal cell carcinoma, pancreatic neuroendocrine tumors, and endolymphatic sac tumors); b) in patients with two or more CNS hemangioblastomas; c) or in patients with a retinal or CNS hemangioblastoma plus at least one visceral tumor characteristic of the disease, excluding renal and epididymal cysts. Imaging plays an important role in the diagnosis and follow-up of patients with VHL disease. This pictorial essay presents characteristic images of abdominal manifestations of VHL disease-related tumors that all radiologists should be aware of.

## Abstract (pt)

A doença de von Hippel-Lindau (VHL) é uma desordem autossômica dominante monogênica com mutações na linha germinativa do antioncogene VHL, no braço curto do cromossomo três (3p25-26). Afeta 1:36.000-50.000 indivíduos, com penetrância superior a 90% aos 65 anos de idade. Embora tenha início e apresentação variáveis, com pleiotropismo mesmo entre membros da mesma família que partilham uma mutação específica, usualmente manifesta-se de início em adultos jovens e predispõe ao desenvolvimento de tumores benignos e malignos no sistema nervoso central (SNC) e órgãos viscerais. Clinicamente, o diagnóstico pode ser realizado em uma das seguintes circunstâncias: a) em pacientes com história familiar de doença de VHL e pelo menos um dos tumores característicos relacionados à síndrome (como hemangioblastomas retinianos ou do SNC, carcinoma de células renais de células claras, tumores neuroendócrinos pancreáticos e tumores do saco endolinfático); b) dois ou mais hemangioblastomas do SNC; c) um hemangioblastoma retiniano ou do SNC mais pelo menos um tumor característico visceral relacionado à síndrome, excluindo-se cistos renais e epididimários. Nesse contexto, a imagem ocupa importante papel no diagnóstico e acompanhamento desses pacientes. Este ensaio iconográfico apresenta imagens características de manifestações abdominais de tumores relacionados à doença de VHL que todos os radiologistas devem conhecer.

## INTRODUCTION

Von Hippel-Lindau (VHL) disease is a hereditary multiple neoplasia syndrome in which patients are predisposed to the development of benign or malignant, synchronous or metachronous, hypervascular tumors and cysts of the central nervous system (CNS) or visceral organs^(1)^. The name refers to Dr. Eugene von Hippel and Dr. Arvid Vilhelm Lindau. Dr. von Hippel was a German pathologist and ophthalmologist who, in 1904, described in detail a rare disease called “retinal angiomatosis”. Dr. Lindau was a Swedish neuropathologist and bacteriologist, whose 1926 doctoral thesis addressed the association between cerebellar hemangioblastomas and retinal angiomas, as well as renal, pancreatic, and epididymal lesions, which would form the basis of much of the modern study of the disease. The gene responsible for VHL disease was identified in 1993, and the disease is the main cause of hereditary renal cell carcinomas (RCCs) and pheochromocytomas^(1)^. Clinically, VHL disease can be classified by clinical phenotype, each phenotype correlating with a specific genotype^(1)^: type 1-low risk for pheochromocytoma but high risk for hemangioblastomas, clear cell renal carcinoma, cysts, and pancreatic neuroendocrine tumors; type 2A-high risk for pheochromocytoma but low risk for clear cell renal carcinoma; type 2B-high risk for pheochromocytoma and clear cell renal carcinoma; and type 2C-high risk only for pheochromocytoma. In this pictorial essay, we focus on the imaging aspects of the abdominal manifestations of VHL disease.

## RENAL MANIFESTATIONS

Multicentric renal cysts and RCCs of the clear cell histological subtype are seen in more than two-thirds of patients with VHL disease^(1)^. The RCCs associated VHL disease tend to develop at an earlier age than do sporadic RCCs (35-40 vs. 55-60 years of age), and the former are often bilateral and multicentric^(1,2)^. The cystic lesions can be a combination of simple cysts, atypical complex cysts, and cystic RCCs^(2)^. In patients with VHL disease, all renal lesions, including simple cysts, should be monitored by imaging because of their malignant potential. There is no correlation between size and malignant potential. The rate of growth and any sign of transformation from cystic to solid (septations, solid components, or contrast enhancement) should be carefully evaluated because they can be indicative of tumor progression. Computed tomography (CT) and magnetic resonance imaging (MRI) are often used to improve the assessment of kidney lesions suspected of being RCCs, as well as in the staging of such lesions.

On CT, RCCs tend to be heterogeneous or show intense early contrast enhancement and progressive washout. On the basis of the MRI aspects, any enhancement identified on CT can be categorized as pseudoenhancement. Pseudoenhancement refers to an artifactual increase of 10 HU or greater over the attenuation of simple renal cystic lesions in the nephrographic phase of CT. An increase of less than 10 HU is considered to be within the limit of normal and is not categorized as enhancement. Pseudoenhancement can occur due to technical variations, such as the use of a greater number of detectors, and anatomical variations, such as a smaller lesion, a more central location in the kidney, and the part of the cyst measured, as well as the adjacent renal and extrarenal structures^(3)^.

**Figure 1:**
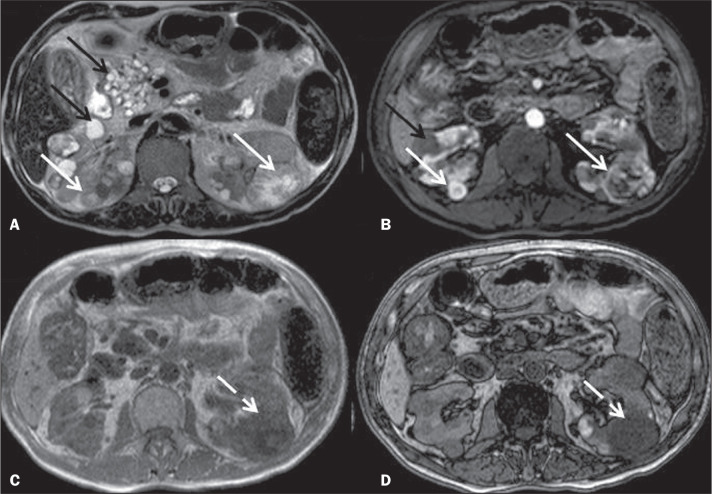
Axial MRI. T2-weighted sequence (**A**) and T1-weighted sequence in the corticomedullary phase (**B**), together with in-phase and out-of-phase T1-weighted gradient-echo sequences (**C** and **D**, respectively), showing multiple pancreatic cysts, in both kidneys (black arrows), some containing blood and having a high protein content, presenting as hypervascular, heterogeneous solid nodules (solid white arrows), with an isointense or hyperintense signal on T2-weighted images. In the out-of-phase sequences, note the signal drop (dashed white arrow), reflecting the presence of microscopic fat, in the nodule within the left kidney. Taken together, these findings are characteristic of clear cell RCCs in a patient with VHL disease.

On MRI, although the visual identification of intralesional enhancement may be sufficient for its characterization, a ≥ 15% increase in signal intensity in relation to the unenhanced T1-weighted images can be considered suggestive of RCC. Even simple cystic lesions can present an increase in signal intensity of up to 5% after contrast agent injection, probably due to movement artifacts or partial volume^(3)^. The term “clear cell(s)” refers to the microscopic accumulation of glycogen and fat. This microscopic fat on MRI can promote a drop in the signal intensity on out-of-phase T1-weighted images (Figure 1). Due to its potential for metastasis, RCC is the main cause of death associated with VHL disease. The objective of imaging surveillance and therapy is to eliminate such lesions before secondary involvement occurs. Because RCC metastasizes to the liver, lung, bone, pancreas, CNS, and epididymis, it is necessary to make the differential diagnosis with tumors characteristic of VHL disease, such as pancreatic neuroendocrine tumors, CNS hemangioblastomas, and epididymal cystadenomas.

## PANCREATIC MANIFESTATIONS

**Figure 2:**
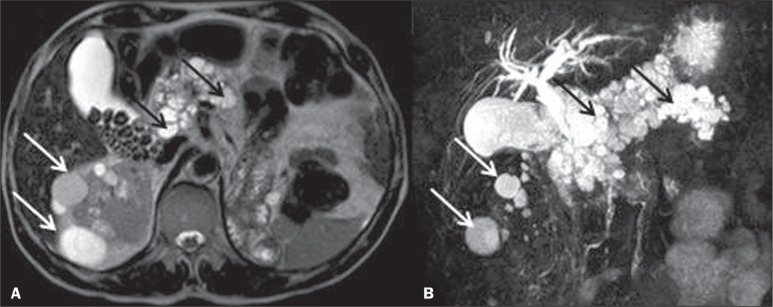
MRI. Axial T2-weighted sequence (A) and three-dimensional cholangiography (B) in a patient with VHL disease, showing that the pancreatic parenchyma had been replaced by numerous diffusely sparse cysts (black arrows). There are also multiple renal cysts, some simple and others with hemorrhagic and high protein content (white arrows). Additional finding: gallbladder stones (A).

Among patients with VHL disease, pancreatic cysts develop in 42%, whereas serous cystadenomas and pancreatic neuroendocrine tumors develop in 11% and 15%, respectively^(1,2,4)^. Such pancreatic cysts are usually multiple and appear as hypoattenuating lesions without contrast enhancement (Figures 1 and 2). Pancreatic cysts may be the only manifestation of VHL disease. If they are too numerous, the patients may develop diabetes; if they are too bulky, they may obstruct the pancreatic duct.

**Figure 3:**
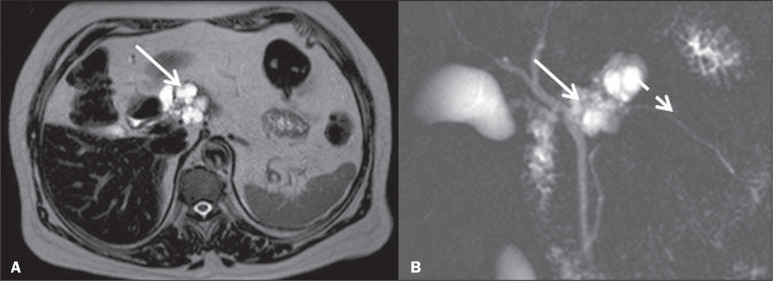
Microcystic adenoma (serous cystadenoma) of the pancreas. T2-weighted axial MRI sequence (A) and MRI cholangiography (B), showing a multicystic lesion, composed of clustered microcysts (“honeycomb” appearance), with a fibrotic scar (hypointense signal on T2-weighted imaging) and lobulated contours, without solid components or ductal communication (solid white arrow). Note the lack of dilation of the main pancreatic duct (dashed white arrow).

Serous cystadenomas manifest as septated, multiloculated cystic masses. They are benign epithelial lesions that form cysts (six or more) measuring up to 2.0 cm each, usually smaller than 1.0 cm. The walls and septa are thin, less than 2 mm thick, with contrast enhancement. On MRI, serous cystadenomas produce a signal that is typically hyperintense on T2-weighted images and hypointense on T1-weighted images, although it can be hyperintense on both if there is intracystic hemorrhage. A fibrotic central scar, when present, produces a hypointense signal on T1- and T2-weighted images, together with delayed contrast enhancement. Despite being pathognomonic, a central scar, with or without calcification, is seen in only 20-30% of cases and in even fewer cases if the lesions are small. In the absence of a scar, the combination of a microcystic appearance and vascular contrast enhancement suggests the diagnosis. Serous cystadenomas do not communicate with the pancreatic duct (Figure 3).

**Figure 4:**
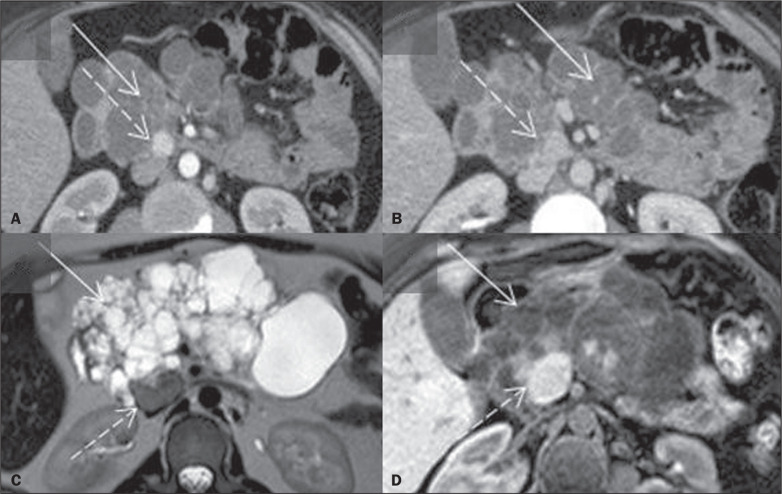
Contrast-enhanced axial CT scan, in the arterial and nephrographic phases (A and B, respectively), together with a T2-weighted MRI scan (C) and a contrast-enhanced T1-weighted MRI scan, in the arterial phase (D), showing enlargement of the pancreas due to multiple, thin cystic wall lesions (solid white arrow), which have replaced the pancreatic parenchyma, in a patient with VHL disease. Note the well-defined, hypervascular solid lesion in the uncinate process (dashed white arrow) on CT, which may be difficult to visualize in phases other than the arterial phase. On MRI, in addition to arterial hypervascularity, the lesion can be characterized by the hypointense signal on T2-weighted imaging and by the absence of ductal communication.

Pancreatic neuroendocrine tumors develop in 9-17% of patients with VHL disease. Compared with sporadic pancreatic neuroendocrine tumors, those associated with VHL disease manifest earlier (mean, 35 vs. 58 years of age). The neuroendocrine tumors seen in VHL are typically multifocal, most commonly being located in the pancreatic head and uncinate process. Although most sporadic and VHL disease-related pancreatic neuroendocrine tumors are nonfunctioning (70% and 98%, respectively), there can be abdominal pain, weight loss, jaundice, pancreatitis, and, more rarely, gastrointestinal bleeding^(1,4)^. The clinical and biochemical characteristics of functioning tumors vary depending on the polypeptide produced. On unenhanced CT, pancreatic neuroendocrine tumors are usually hypoattenuating or isoattenuating. On contrast-enhanced CT and MRI scans, such tumors show intense (homogeneous, annular, or heterogeneous) contrast enhancement early in the arterial phase, often showing enhancement identical to the rest of the pancreas in the other phases, which calls for rigor on the part of the radiologist when a specific diagnostic protocol is adopted. Pancreatic neuroendocrine tumors do not have a direct relationship with the ductal system. In patients with VHL disease, pancreatic neuroendocrine tumors are often diagnosed on the basis of imaging alone^(1,4)^, as depicted in Figure 4.

## OTHER MANIFESTATIONS

### Pheochromocytomas and paragangliomas

**Figure 5:**
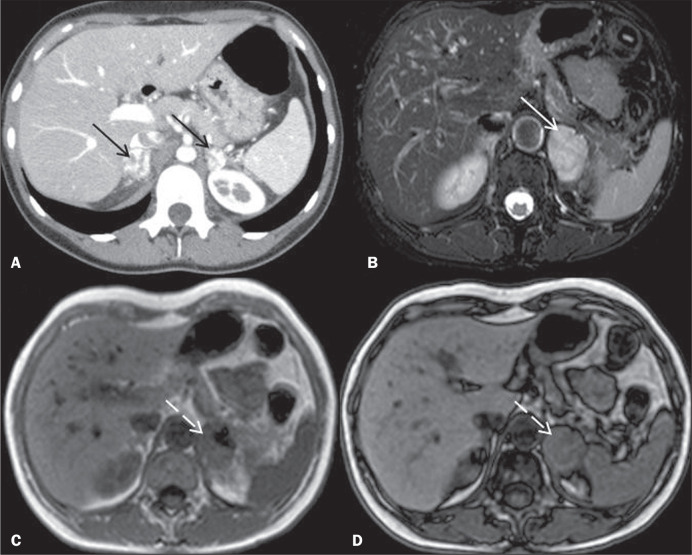
Contrast-enhanced axial CT scan in the arterial phase (A), showing lesions with intense heterogeneous enhancement in both adrenal glands (black arrows) in a patient with elevated levels of fractionated plasma metanephrines, consistent with pheochromocytomas. In another patient with VHL disease, T2weighted spectral presaturation with inversion recovery MRI sequence (B), together with in-phase and out-of-phase T1-weighted gradient-echo sequences (C and D, respectively), showing a lesion in the left adrenal gland with high signal intensity on the T2-weighted image (solid white arrow) and absence of signal loss in out-of-phase sequences (dashed white arrow), findings typical of a pheochromocytoma.

**Figure 6:**
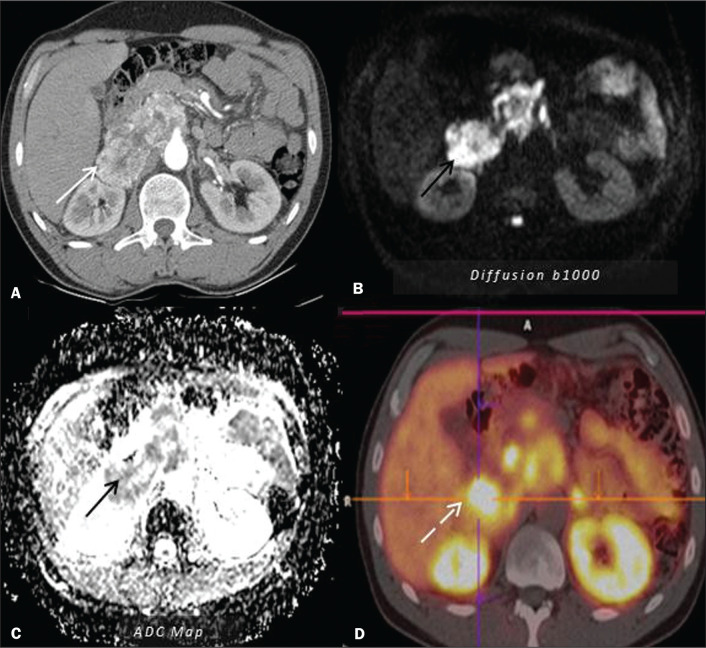
Paraganglioma. CT (A) and MRI (B,C), showing an expansile, solid lesion in the right, retroperitoneal para-aortic region. The lesion was hypervascular in the arterial phase on CT (white arrow in A) and showed high signal intensity on T2-weighted MRI scans, without microscopic or macroscopic fat foci, with restricted diffusion on diffusion-weighted imaging (black arrows in B and C). Positron-emission tomography/CT with gallium-68 dotatate (D), showing intense avidity of the radiotracer for the lesion (dashed arrow).

Among patients with pheochromocytomas or paragangliomas, the production of catecholamines (epinephrine, norepinephrine, and dopamine) is highly variable. Therefore, the clinical presentation can range from asymptomatic to sudden death. Such patients may present with the triad of headache, diaphoresis, and tachycardia associated with arterial hypertension. Normotensive, asymptomatic patients account for 5-15% of cases of pheochromocytoma or paraganglioma. The diagnosis is based on evidence of the production of catecholamines and their metabolites, the initial diagnostic tests including determination of the levels of metanephrines in urine and plasma, with more specific assessment of plasma fractions in hereditary cases. Pheochromocytomas and paragangliomas associated with VHL disease produce mainly norepinephrine^(1,4)^. They can occasionally cause paraneoplastic syndromes (most commonly Cushing’s syndrome, due to ectopic production of adrenocorticotropic hormone). Pheochromocytomas are seen in 25-30% of cases of VHL disease, whereas paragangliomas are seen in 15%, the latter being found along the sympathetic chain in the abdomen, chest, head, and neck^(5,6)^. Like their clinical presentation, their imaging manifestation is varied. The lesions are mostly solid and heterogeneous but can have cystic areas. They typically present intense enhancement after contrast administration, which reveals them to be hypervascular, mainly in their solid components^(1,2)^. It should be borne in mind that the absolute or relative washout of pheochromocytomas on CT may overlap with that of an adenoma or a malignant lesion; therefore, contrast-enhanced CT may not necessarily facilitate the diagnosis^(7,8)^. The high signal intensity of a pheochromocytoma on T2-weighted MRI scans-the so-called light bulb sign-is a useful feature for the diagnosis, despite the fact that it is present in only 11-65% of cases, because a portion of the lesion can be intermingled with a cystic component^(9)^, as illustrated in Figure 5. On T1 weighted MRI scans, pheochromocytomas show a signal that is usually isointense but can be hyperintense if there is hemorrhage. Albeit rare, intracellular fat can be seen in the lesion, which results in a loss of signal intensity on outof-phase T1-weighted images, as in adenomas. As a consequence of the different degrees of pathological degeneration, pheochromocytomas can present a broad spectrum of imaging presentation (which has made them known among radiologists as “chameleon tumors”). Because of the broad spectrum of imaging manifestations, pheochromocytomas and paragangliomas often need to be evaluated by functional studies for greater diagnostic accuracy and to detect extra-adrenal or metastatic disease^(10)^, as shown in Figure 6. In comparison with pheochromocytomas, sympathetic paragangliomas present a higher risk of metastasis^(11)^. The evolution of metastatic disease varies from case to case: most patients with metastatic pheochromocytoma or paraganglioma survive for 2-4 years, although some survive for 20 years or more. In view of such prognostic differences in metastatic disease and the temporal unpredictability of onset, such patients should be followed over the long term.

Bilateral papillary cystadenomas of the epididymis should raise strong suspicion of VHL disease, given that 60% of such tumors are attributed to the disease. A papillary cystadenoma of the epididymis usually measures ≤ 4.0 cm. The presentation on ultrasound ranges from a cystic mass with hypoechoic content to a predominantly solid mass, usually with increased flow on color Doppler. The vas deferens may be dilated. Cystadenomas rarely occur in the broad ligament of the uterus or in the mesosalpinx^(1)^.

## CONCLUSION

Knowledge of the main imaging findings of VHL disease can empower radiologists to establish associations in cases in which the findings are suggestive of the syndrome, allowing them to make the initial diagnosis of previously unknown cases, with an emphasis on the lower range of the age of onset of many of the associated lesions. In addition to the initial diagnosis, abdominal imaging plays an important role in the screening/early detection and followup of the lesions (some with higher risk than others), in accordance with the follow-up protocols proposed^(12-14)^. Together with multidisciplinary groups and medical teams, radiologists seek better care for patients with VHL disease, which could improve their quality of life and reduce the morbidity and mortality associated with the disease.

## References

[1] Ganeshan D, Menias CO, Pickhardt PJ (2018). Tumors in von Hip-pel-Lindau syndrome: from head to
toe-comprehensive state-ofthe-art review. Radiographics.

[2] Schwingel R, Duarte SBL, Oshima MM (2015). Which is your diagnosis?. Radiol Bras.

[3] Tappouni R, Kissane J, Sarwani N (2012). Pseudoenhancement of renal cysts: influence of lesion size,
lesion location, slice thickness, and number of MDCT
detectors. AJR Am J Roentgenol.

[4] Gläsker S, Vergauwen E, Koch CA (2020). Von Hippel-Lindau dis-ease: current challenges and future
prospects. Onco Targets Ther.

[5] Choi YA, Kim CK, Park BK (2013). Evaluation of adrenal metastases from renal cell carcinoma and
hepatocellular carcinoma: use of delayed contrast-enhanced
CT. Radiology.

[6] Aufforth RD, Ramakant P, Sadowski SM (2015). Pheochromocytoma screening initiation and frequency in von
Hippel-Lindau syndrome. J Clin Endocrinol Metab.

[7] Blake MA, Kalra MK, Maher MM (2004). Pheochromocytoma: an imaging chameleon. Radiographics.

[8] Schieda N, Alrashed A, Flood TA (2016). Comparison of quantita-tive MRI and CT washout analysis for
differentiation of adrenal pheochromocytoma from adrenal
adenoma. AJR Am J Roentgenol.

[9] Jacques AET, Sahdev A, Sandrasagara M (2008). Adrenal phaeo-chromocytoma: correlation of MRI appearances with
histology and function. Eur Radiol.

[10] Prasad V, Tiling N, Denecke T (2016). Potential role of (68)Ga-DOTATOC PET/CT in screening for
pancreatic neuroendocrine tumour in patients with von Hippel-Lindau
disease. Eur J Nucl Med Mol Imaging.

[11] Turchini J, Cheung VKY, Tischler AS (2018). Pathology and genetics of phaeochromocytoma and
paraganglioma. Histopathology.

[12] Mourão JLV, Borella LFM, Duarte JA (2022). Imaging manifestations of von Hippel-Lindau disease: an
illustrated guide focusing on the central nervous system. Radiol Bras.

[13] VHL Alliance (2020). The VHL handbook: What you need to know about VHL.

[14] Poulsen MML, Budtz-Jørgensen E, Bisgaard ML (2010). Surveillance in von Hippel-Lindau disease (vHL). Clin Genet.

